# Evidence-Based Study to Compare* Daodi* Traditional Chinese Medicinal Material and Non-*Daodi* Traditional Chinese Medicinal Material

**DOI:** 10.1155/2018/6763130

**Published:** 2018-01-22

**Authors:** Xingyue Yang, Xin Tian, Yannan Zhou, Yali Liu, Xinlong Li, Tingting Lu, Changhe Yu, Liyun He

**Affiliations:** ^1^Beijing University of Chinese Medicine, Beijing 100700, China; ^2^State Key Laboratory Breeding Base of Dao-Di Herbs, China Academy of Chinese Medical Sciences, Beijing 100700, China; ^3^The First Clinical Medical College of Lanzhou University, Lanzhou 730000, China; ^4^Institute of Basic Research in Clinical Medicine, China Academy of Chinese Medical Sciences, Beijing 100700, China; ^5^Evidence-Based Medicine Center, School of Basic Medical Sciences, Lanzhou University, Lanzhou 730000, China; ^6^Department of Tuina and Pain Management, Dongzhimen Hospital, Beijing University of Chinese Medicine, Beijing 100700, China

## Abstract

**Background:**

* Daodi* medicinal material is widely used in Chinese herb medication. However, there is a lack of systematic methodology for identifying characteristics associated with good quality and reliable efficacy of* Daodi* med-material.

**Purpose:**

The purpose of this study is to provide some evidence to further substantiate the use of* Daodi* medicinal materials.

**Methods:**

Seven relevant databases were searched before July 2014. Two evaluators were responsible for screening and categorizing the results. The data was analyzed with Microsoft Excel 2007 and SPSS 21.0 statistical software.

**Results:**

Overall, 107 articles were systematically analyzed. Of these studies, 55.1% (59/107) focused on the methodology to assess* Daodi* med-material, and 38.3% (41/107) were interested in med-material ingredients, soil physical and chemical properties, and the geological background system (GBS). Only 6.5% (7/107) of studies were mainly conducted as clinical trials and animal experiments.

**Conclusion:**

Comparisons between* Daodi* and non-*Daodi* materials have been studied mainly in terms of the ingredients or composition of medical materials, soil physics and chemistry, and the GBS, and some identifying methodologies have been created to identify* Daodi* attributes. Until now, there is still no consensus of comparison criteria between* Daodi* and non-*Daodi* medicinal material. Only a few studies were conducted through animal experiments and clinical trials to determine* Daodi* superiority.

## 1. Introduction

The term* “Daodi”* medicinal material has often been used in Chinese ethnopharmacology, and it is usually defined as a material that has been screened after a long period of traditional medical practice, growing in a specific region, and associated with a unique production method. Thus, materials with this label are recognized as having high quality and being clinically effective, and they are a reputable hallmark compared to other medicinal materials that are non-*Daodi* and so forth [1].

The Chinese word* “Daodi”* accentuates some distinctive higher quality for the medicinal material that grows in a certain area. The pristine exploration can be retraced to the late Eastern Han Dynasty (25–220 CE) with the advent of the earliest Chinese medicine document* Divine Husbandman's Classic of Materia Medica *(Shennong Ben Cao Jing). In the Tang Dynasty (618–907 CE), the famous scholar Sun Simiao (the renowned Medicinal Material King) proposed in his classic work* Thousand Gold Pieces (Qian jin fang)* categorizing the original region for producing medicine in the contemporary administrative province and emphasized a concept that medicinal material is essentially embedded in soil. Sun Simiao first used the term* “Dao,”* which was the rudimentary concept that later became known as* “Daodi.”* The* “Daodi”* concept was initially defined in the Chinese medical classic* Essentials of Materia Medica Distinctions (Ben cao pin hui jing yao)* in the age of the Ming Dynasty (1368–1644 CE) [2]. The medicinal selection methods had in fact been gradually formulated during medical practice for thousands of years and were established as a unique way to identify materials. Being supported by profound Chinese medical theory, this method is still significant in modern times. Furthermore, the* Daodi* materials are the most thoroughly investigated materials, and they represent a large amount of the market with tremendous economic value. It is reported that there are 200* Daodi* materials out of the 500 traditional medicinal materials, yet* Daodi* materials contribute approximately 80% to the overall usage [3].

Chinese herbal medicine has been used in China for over 2000 years [4]. In traditional Chinese medicine (TCM),* Daodi* materials are considered to have high medicinal efficacy [5]. Most of the studies comparing* Daodi* and* non-Daodi* materials were from China. The theory and practice have been developed over thousands of years. Considering territory distribution,* Daodi* medicinal material can be categorized into Chuan- (Sichuan) Guang- (Guangdong/Guangxi) Yun- (Yunnan) Gui (Guizhou), Nan (Southern China) Bei- (Northern China) Zhe (Zhejiang) Huai (Henan), and Shan- (Shanxi) Gan- (Gansu) Qing- (Qinghai) Ning (Ningxia). Each medicinal material is rooted in locations with optimal breeding conditions. Because it is the delicate complexity in a natural land that endows a* Daodi* material with its perplexing mechanism, it has been conventionalized, probably for an expedient solution, through the use of empiricism to differentiate* Daodi *and* non-Daodi* materials. As a result, although* Daodi* medicinal materials are highly valued and renowned nationwide, until now, there has been a lack of sufficient evidence to corroborate traditional practices during* Daodi* material selection and to identify the superiority, in terms of either quality or clinical efficacy [6].

The purpose of this study is to provide some evidence to further substantiate the perspective on* Daodi* medicinal materials. We searched the relevant modern literature to analyze and assess comparative studies of* Daodi* and non-*Daodi* materials.

## 2. Methods

### 2.1. Inclusion/Exclusion Criteria

Studies that compared* Daodi* Chinese traditional medicinal material and* non-Daodi* traditional medicine were retrieved and included. Review articles were excluded. Article abstracts that could not be traced to their source data and full-text were also ineligible.

### 2.2. Search and Retrieval Strategy

Seven databases were searched, including PubMed, EMBASE, Web of Science, the Chinese Biomedical Literature Database (CBM), the Chinese Journal Full-Text Database (CJFD), the Chinese Scientific Journal Full-Text Database (CSJD), and the Wanfang database. The key words included “famous-region drug”, “authentic medicinal Medicine”, “genuine medicinal material”, “genuine crude drugs”,* “Daodi”* (道地), and “Didao” (地道).

#### 2.2.1. Screening Procedure

Two evaluators (Xin Tian and Yannan Zhou) screened the articles independently by reviewing the title and the abstract. If they reached an agreement that the article met the literature identification standard, the full-text version was sought. Any disagreement was settled by a third evaluator, Yali Liu.

#### 2.2.2. Data Retrieval and Analysis

The data of general characteristics and the overall study/processing report were collected. Two evaluators screened data from each article independently, and any discordance from the search results was discussed or another evaluator consulted to reach a resolution (Yali Liu).

A retrieval form was designed according to the study strategy, which mainly included (1) basic information, such as publication journal and time, academic institution, and study funding and (2) data to compare the* Daodi* and* non-Daodi* medicinal materials for possible differences: ① clinical trials and animal experiments that included basic medicinal material information, methodology, results, and conclusions; ② medicinal material ingredients, the geological background system (GBS), and different chemical and physical soil properties; and ③ a study methodology for identifying a* Daodi* material, including general med-material information, methodological contrast, and other aspects. The producing regions of the* Daodi* and* non-Daodi* described in the basic information and the sample size in either a clinical trial or an animal experiment were recorded as numbers. The following information was described in the form for data collection: growth mode and material sourcing in the basic information section; the reported dose formation and the dosing methods, as well as ingredients, during clinical trials and animal experiments; and other information needed for morphological comparisons between the materials, for the GBS and for a description of the physical and chemical properties and their final comparison conclusion. The other items or contents were recorded as 1 (described in the article) or 0 (not described in the article).

The data were summarized and analyzed by Microsoft Excel 2007 and SPSS 21.0 statistical software, and all the results were described statistically (frequency and percentage).

## 3. Results

### 3.1. Search and Retrieval Outcomes

**Figure 1:**
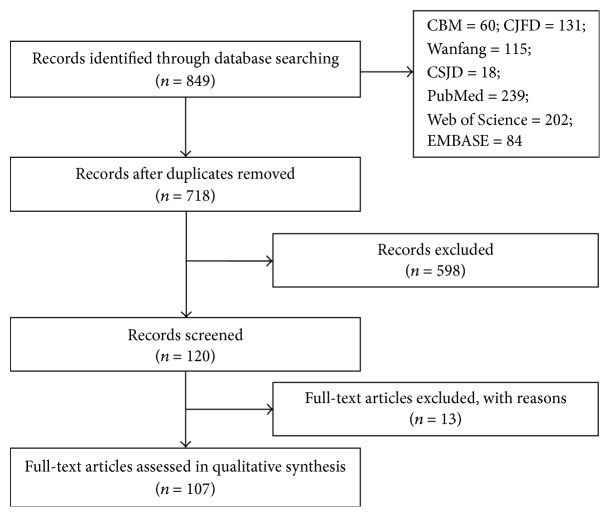
Flow chart of study inclusion.

Overall, 849 articles were originally acquired, including 324 written in Chinese and 551 articles in English. After deleting repetitive or redundant information, screening was implemented after perusing the abstract section, 120 articles were consequently confirmed through the literature identification criteria, and the 120 full-text articles were then evaluated. After scrutiny of the full text, another 13 articles were excluded from the study. The remaining 107 articles were included, including 4 studies in English (Figure 1).

### 3.2. Basic Characteristics of the Included Articles

#### 3.2.1. Basic Information of Included Studies

This study included 67 (67/107) journal theses, 32 (32/107) academic degree dissertations (30 Master's degree dissertations and 2 Ph.D. dissertations), 5 (5/107) conference papers, and 3 published conference recordings. Ten (10/107) authors, constituting the maximal proportion, were scholars from the China Academy of Chinese Medical Sciences. Of the studies, 58.9% (63/107) had a funding source, the other 41.1% (44/107) had no funding support, and 72.7% (32/44) were academic degree dissertations. The four English studies reported that there were no conflicts of interest, and not any claims of interest conflict were written in the Chinese articles. Of the 67 journals, the Chinese Journal of Chinese Materia Medica was the most proportional one at 23.9% (16/67) (Table 1).

The first publication about a* Daodi* medicinal materials and non*-Daodi* comparison study was issued as* Zhong Yao Cai* in 1990. However, from 1990 to 1999, only 3 studies were published, including two studies about traceable mineral elements; the other was a clinical trial. After 2000, there was a continuous increase in the number of relevant studies. The first genetic comparison of the two types of material was published in 2001, and also in this year an animal experiment to investigate the difference in the two types of material was performed. Studies pertaining to the physical and chemical properties of the soil began in 2002. A comparison of med-material ingredients, soil physical and chemical properties, and the GBS was initiated.

#### 3.2.2. Study Categorization

Six different categories were summarized in Table 2, including 1 clinical trial, 6 animal experiments, 2 articles pertinent to the GBS and physical or chemical properties of the soil, 24 articles that exclusively investigated medicinal ingredients, 15 articles involving the medicinal composition, the GBS, and the chemical or physical properties of the soil, and 59 articles seeking a methodology to assess non-*Daodi* materials.

A total of 24 med-materials were included in this investigation and published in the 107 articles. The investigated med-materials were listed below: Pheretima Aspergillum (the only material with a zoological origin), Morindae Officinalis Radix, Atractylodis Rhizoma, Citri Reticulatae Pericarpium, Carthami Flos, Chuanxiong Rhizoma, Rhei Radix et Rhizoma, Salviae Miltiorrhizae Radix et Rhizoma, Moutan Cortex, Angelicae Sinensis Radix, Codonopsis Radix, Rehmanniae Radix, Poria, Aconiti Lateralis Radix Praeparata, Polygoni Multiflori Radix, Magnoliae Officinalis Cortex, Coptidis Rhizoma, Scutellariae Radix, Astragali Radix, Lonicerae Japonicae Flos, Ophiopogonis Radix, Cyathulae Radix, Ginseng Radix et Rhizoma, Notoginseng Radix et Rhizoma, Dioscoreae Rhizoma, Paeoniae Radix Alba, Himalaica Mirabilis, Asari Radix et Rhizoma, Scrophulariae Radix, Polygalae Radix, Alismatis Rhizoma, Anemarrhenae Rhizoma, Aurantii Fructus, and one from a mineral (Gypsum Fibrosum).

### 3.3. Comparison of* Daodi* Medicinal Materials

#### 3.3.1. General Information of the Origins and Identification of* Daodi* Medicinal Materials

Because the production regions were divided according to rather inconsistent definitions in these studies, we described producing regions as more than one and only one. More than half of the studies did not describe the growth mode. The majority of the studies (78.5%, 84/107) provided information about the process of acquiring med-materials. For example, procurement from a certain Chinese traditional medicine market or going to the original region for purchase was described by some professors or labs. The med-materials were identified by some experts in only 35.5% (38/107) of the articles. No study specified identification methods in detail. Some studies only mentioned that the med-material was identified; however, there was often no record of any professional assigner, and in some cases, the material extraction process was erroneously considered as the identification process. Of the studies, 52.3% (56/107) described specific timing about natural med-material reaping (at least roughly for a specified lunar-month). Most of the studies described the basic properties, pharmacological activity, and/or documented efficacy of the Chinese medicine (Table 3).

#### 3.3.2. Data from Clinical Trials and Animal Experiments

Only 6 studies were designed with animal experiments to compare* Daodi* and* non-Daodi* materials. One clinical trial investigated differences in the effectiveness between the two material types. The first clinical study was published in 1990, and it compared the effectiveness and safety between rhubarbs.* Daodi* or* non-Daodi* materials were used to treat upper gastrointestinal hemorrhage, and the results demonstrated that the* Daodi *rhubarb had a more effective cure rate and reduced adverse events. Most of the animal experiments in both mice and rats (83.3%) were published after 2010, with 16.7% (1/6) describing* in vitro* experiments and 83.3% (5/6) describing* in vivo* experiments. The sample size varied from 42 to 140 (mean 96). All the* in vivo* experiments included randomization into groups, and an aqueous extract was administered most often. All these studies reported pharmacodynamics results, and there were no adverse events. The majority of these studies (83.3%) reported that the* Daodi* materials were more effective than the non-*Daodi* materials, and 25% reported that the two med-materials had similar effective results.

#### 3.3.3. Medicinal Material Ingredients, Soil Properties, and the GBS

Forty-one studies investigated the med-material ingredient, soil properties, and GBS. Two studies compared the GB and soil properties, 24 studies were exclusively about the material ingredients, and 15 articles included all the subjects mentioned above. Three articles examined the different arable soils (different region) that were selected to raise the same herbs, and the possible effect of changes in some inorganic or mineral elements could be analyzed. In this review, we put this article in the category of covering all subjects. For expediency, we added associated study subjects, which included endophyte microbes and metabolites, morphological contrast, and germplasm resource and genetic analysis, Table 4.

Med-material ingredient comparisons were performed mostly to examine mineral and organic compositions. Of these, 63.4% (26/41) compared the active ingredients of an organic composition, and one study investigated pesticide residue, heavy metal substances, and the ineffective ingredients (phenylformic acid) of two med-materials. Overall, 58.5% (24/41) analyzed mineral element discrepancies, and 4.9% (2/41) reported the probable influence on pharmacodynamics from these minerals. For organic ingredient comparison, many of the studies were focused on the active component of med-materials. The difference of volatile oil between Atractylodis Rhizoma and Chuanxiong Rhizoma was the most frequently analyzed, accounting for approximately 38.5% of studies (10/26). Some studies examined extracts, polysaccharides, and total ash proportions as the main discrepant indicators between the* Daodi* and* non-Daodi *materials. Morphological contrasts were presented in 26.8% (11/41) of the studies, including physiological anatomy or functional observation. Two studies were conducted to contrast the microanatomy, and one observed some ultramicrostructures.

Some scholars observed the constituents, soil features, and GBS to try to find possible differences in certain med-materials. Mineral quantities were analyzed in 36.6% (15/41) of the studies, and 22.0% (9/41) examined the pH of soil samples. Most GBS studies were conducted to determine geographic characteristics of growing locations, such as altitude and latitude. There were also considerable studies about climatological parameters (i.e., type of climate or conditions, annual sunshine duration, rainfall, and temperature) and the geological background (topography and landforms). A few studies (2.4%, 1/41) addressed concerns about wind velocity and the humidity of different breeding places (Table 4).

#### 3.3.4. Methods for Identifying* Daodi* Med-Material

Most of the methods used to define a* Daodi* med-material have depended on physics, chemistry, and biological techniques, as well as statistics, to distinguish apparently similar natural materials, or different production, processing, or concoction procedures have been analyzed to further investigate effective components that might have been affected by processing (Table 5). Fifty-five studies described a methodology to identify a* Daodi* material, and most of these involved a fingerprint technique. These techniques included chromatography, spectrometry, MRI (magnetic resonance imaging), and DNA fingerprint. Thin-layer chromatography (TLC), high-performance liquid chromatography (HPLC), and gas chromatography (GC) were used. Diode array detector (DAD), Fourier Transform Infrared (FT-IR), and Near Infrared (NIR) were used for spectrometry. MS was the only MRI analysis. HPLC has been the most frequently used approach, with 50.8% (30/59), and the other widely used techniques included GC-MS and HPLC-DAD. Fingerprint DNA was reported in 11 studies. The profile of fingerprint DNA in* Daodi *med-materials was established in 37.3% (22/59) of the included studies. Cluster analysis was the most frequent method for statistical analysis (52.5%,31/59). Another 8.5% (5/59) included principal component analysis (PCA) processing. Five (8.5%) studies investigated a process to prepare, produce, and concoct a medicine, which might also contribute to identifying the quality of* Daodi*.

#### 3.3.5. Results and Clinical Practices

Several studies were investigated in this review. Of these studies, 64.5% (69/107) claimed that* Daodi* med-materials were superior to* non-Daodi *med-materials. Only a small number, 1.9% (2/107), suggested that statistical significance could not be found. Additionally, 2.8% (3/107) of these studies analyzed the active ingredients of the materials and discovered that the* Daodi *med-materials had fewer active ingredients. Another 30.8% (33/107) did not make any comparison of superiority or inferiority between the two types of material (Table 6).

## 4. Discussion

### 4.1. Complicated Environments of* Daodi* Breeding and Incomplete Studies according to the Identification of a* Daodi* Med-Material

Previous studies about* Daodi* med-materials mostly involved medical material tests [7–9], the foundation for formation and development [7, 10–12], quality evaluation and identification/verdicts [13–16], and GAP development [17–22]. In addition, these approaches had mainly been conducted through review and experiment studies. The first study about comparisons was published approximately 20 years ago, and pertinent studies have been increasing gradually. To the best of the author's knowledge, this study is the first review that presents a comparison between* Daodi* and* non-Daodi* med-materials.

The pertinent studies on the comparison of* Daodi* and* non-Daodi *materials were first published in the 1990s. Beginning in 2000, well-rounded development began again, except for contrasting the ingredients or compositions and pharmacodynamics; other aspects, such as soil characteristics, the GBS, the evaluation methods, and animal experiments, were also inclusively conducted. In 2001, the first exploratory study for some* Daodi* and non-*Daodi *genetic comparisons produced a study that has substantiated our molecular insight into these plants. From 2000 to 2010, the published studies were mostly performed through animal experiments, soil analysis, and GBS comparison. Since 2010, the study trends included animal experiments and the introduction of contemporary techniques to identify* Daodi* and non-*Daodi *Chinese traditional medicinal material.

When studying med-material ingredients and composition or soil properties, most often, scholars will compare either mineral or organic ingredients. For example, mineral differences in the two categories of med-materials were usually investigated. However, only a few of these studies established certain mineral characteristic as a significant indicator for claiming authenticity.

The GB is defined as the specific synthesis of attributes in a geologic body and geologic agents that are highly related to the med-materials, including quaternary sediment, the mineral distribution or rock mass, tectonics, crustal movement, geographic and geomorphic factors, topography and landforms, geochemistry, hydrogeology, and other multifarious considerations. In fact, the GBS is defined as a GB 2-dimensional integrality (GB, climate, biology, etc.), which is part of modern system theory about the foundation of natural nonequilibrium open system consistence law [9]. Most of the studies in this review are about the original production regions in terms of their geographic features, climate data, GB, and so on. Regarding differences in pivotal ingredients, the pharmacodynamics of the two types of med-material have high relationships with the geological milieu. Considering the complications in geographic environments, there are remarkable discrepancies in various soil and water conditions, climates, sunlight durations, and distributions of biological beings. All these factors may affect the quality of the med-materials, and as they may play a role in the differences in medical efficacy, these issues require further clinical trials to provide substantial evidence of these effects.

A majority of the studies to identify* Daodi *Chinese traditional medicinal material used a fingerprint method, and HPLC was the second most common approach. Many studies identified the med-materials via only one method for fingerprint examination, and a combination of two methods for fingerprint study was also frequently applied, especially GC-MS and HPLC-DAD [19]. Few studies were facilitated by DNA fingerprint graphing. However, there are great variations in applying specific methods. In addition, NMR was also used in the analysis of plant extracts [20].

### 4.2. The Current Challenges, Issues, and Prospective Solutions

Even among the wide range of data sources sought in this review study to compare the overall investigational results between* Daodi* and* non-Daodi* materials, only 34 med-materials were covered, which contrasts with the more than 200* Daodi* materials that have been recorded. This outcome suggests that other materials have not been sufficiently investigated. Ingredient differences were unanimously considered to be one of the standards for claiming superiority. However, for many studies in this review, a view of the growth mode was not taken for either* Daodi* or non-*Daodi *med-materials; further investigation on specific provenances, sourcing status, and reaping time was not considered; there was no report about whether an experimental identification was conducted between the two types of material; and there was a similar deficiency for articulating the processes and methods to claim a difference. In fact, this information has great significance in regard to comparing the functional ingredients of* Daodi* and non-*Daodi *materials as well as clinical effectiveness. Therefore, it is highly recommended that producing region information be considered for clarification. In cases regarding the necessary identification of the attributes of some med-materials, experts and professionals should be invited to consult. If so, the study's accuracy would be enhanced with reliable and intelligible scientific evidence, which would benefit the development of future translational medical studies.

Suitable geographical conditions are exterior factors for producing* Daodi* ingredients [1]. Admittedly, the Chinese geological environment has evolved on a large scale over time and under natural conditions. As a result, it may play a role in the transference of the* Daodi* med-material producing region. However, the fundamental principle for trustworthiness of* Daodi* med-material has always been stated as “Good Quality and High Efficacy” for its label and “Foundation on the Primacy” [23] for its essence. Two studies [24, 25] included in this review have distinguished the original region using the “ancient” tract and “current” tract, and both are attributed to the* Daodi* category, which disregards a possible effect on the medicine quality when considering the changes in the methods applied for producing region division or categorization.

Until now, clinical discrepancies were derived from empiricism rather than from more substantial trial results. In addition, modern studies still take material ingredients, soil properties, and the GBS as the investigational cynosure. Studies have seldom been performed through animal experiments and clinical practical tests. Furthermore, both of these types of study have only been sparsely and fragmentally reported. In most of the real cases and dosing in Chinese traditional medicine, a combination recipe with different materials was prescribed, and it was seldom administered as just a single medicinal ingredient/dose. Because there are interactions between different medicine ingredients and agents, with different mechanisms that could complicate the results, some scientists consider that even clinical trials, which are a widely accepted study method, cannot be taken as a valuable way to interpret whether any single medicine from different producing areas can be differentiated or confirmed to have an exclusive effectiveness. There might be only some slight or subtle difference between the* Daodi* and non-*Daodi *materials that is not as remarkable as the contrast between a medicinal agent and a placebo in established Western clinical pharmacology. As a result, the study has to include many more subjects to present a difference, which therefore poses a great challenge to obtaining clinical testimony. None of these included studies has investigated toxicity and adverse events, and there is evident incompleteness of data for toxic comparison between the medicinal materials. If the effectiveness of* Daodi* and non-*Daodi *materials in animal models is to be compared, it has been reasonably suggested that some parallel studies with a well-controlled design, as well as with wholeness and precision, be preferably established to investigate the relevant toxicity to further dig deeply into the discovery of the pharmacodynamics and safety profile.

One certain medicinal material may be produced from different regions, and its active ingredients and quantities can vary. Active ingredients or components are a uniquely important attribute for any* Daodi *med-material [23]. However, it is necessary to note here that a quality verdict might not be proportionally related to certain chemical compositions. In fact, certain levels of some ingredients cannot substantially indicate a remarkable or significant difference between one* Daodi* and another non-*Daodi *material [26]. Endophytes are some fungi or bacteria that have been living in healthy plant tissues or organs throughout the plants' whole life or some special growth stage; in addition, endophytes can beneficially play a role in some formation or accumulation of active ingredients [27]. Derived metabolites are usually regarded as the main ingredients or components in Chinese traditional medicine. The benefits of these derived metabolites have been commonly agreed on, such as disease-resistance, antipest, and counter-environmental adversary effects [28]. Only three studies were conducted on endophytes and compared derived metabolites in Chuanxiong Rhizoma and Lonicerae Japonicae Flos to investigate their* Daodi* attributes [29–31].

The superior germplasm resource is an internal factor of the formation of a* Daodi* medicinal material [1], and for this reason, many modern studies compare soil properties, such as mineral differences in producing soil that may breed a* Daodi* or* non-Daodi* material. However, there is still a lack of evidence that necessitates some further studies to investigate the relationships between soil minerals and the medicine toxicity, and the compound minerals that may be formed into any quality, as well as med-material yields, especially indispensable minerals that could improve the quality of traditional medicine, are still an open issue. We suggest that these aspects require further study to clarify the association between soil minerals and the chemical composition of the med-materials as well as to determine if pragmatic significance was involved in the process of cultivation and quality assurance, along with fertilization for time and quantity considerations [32]. All the comparisons of the soil properties and various environmental factors in this study to ascertain significant differences between* Daodi* and non-*Daodi *med-materials are designed to discover natural conditions that formulate and compose* Daodi* supreme qualities. However, there is a lack of objective and clear methods as well as standards to produce reliable contrasts. Therefore, these pertinent studies have unfortunately been bogged down in a perplexing impasse, “purposed for a study, but without any credible measurement” and “wheeling into repetitive inanity.” As a consequence, it is very difficult to disclose any ecological factor to identify a* Daodi* material. From this review, we recognize that assessment methods and standards must be established if the relationship between* Daodi* attributes and the ecological environment is to be studied [26].

Morphological comparison between* Daodi* and* non-Daodi* med-materials is one of the key aspects; however, it often highlights physiological states directly from observations, appearances, or phenomena, which usually come from individual empirical knowledge and are probably prejudiced by different viewpoints subjected to a provincial judgment. Attributed to these incomplete and nonquantified standards, these methods can be applied and developed with limitations.

Recently, new technologies and methods have been developed rapidly to be used for* Daodi* med-material identification, and these approaches will hopefully overcome some difficulties of traditional methods for identifying differences. Shilin Chen suggested that DNA barcoding [33] can be used for med-material sample identification when there is little background information. This method is advantageous for methodological generalization and digitalization as well as developmental feasibility [34]. He also proposed the Herb Genome Project (HerbGP) (Chen et al., 2010) and established a DNA barcoding [35] system, which has great potential. Xiaohe Xiao suggested establishing a “synergic* Daodi* med-material standard” (*Daodi* Indicator, DDI), and this method might be more systematic and objective for a control assessment as well as for confirming high quality med-materials [36] (Xiao et al., 2012). Xueyong Wang proposed some more systematic and precise methods for Chinese traditional medicine study to produce reliable evaluations [37]. In 2002, a complicated systematic theory about the Chinese traditional medicinal material GAP tool was suggested by Luqi Huang [17]. The author applied modern physics, chemistry, biology, and statistical methods to identify ingredients and confirm the quality of two med-materials, even though no agreement was reached.

### 4.3. Limitations of This Study

Above all, only seven Chinese or English databases were included as data sources, and the final studies included were entirely written in Chinese. No English articles were retrieved. Second, this review is only based on modern studies, and no associated provenance from ancient studies has been retraced. The third limitation is that it is possible to miss some studies because the designated keywords have not been included anywhere in an article's title, abstract, and keyword list, especially in cases where there was some specific description with different vocabularies, although this study began with a systematic retrieval method and plan.

## 5. Conclusion

Comparisons between* Daodi* and non*-Daodi* materials have been studied mainly in terms of med-material ingredients or composition, soil properties, the GBS, and some identifying methodologies to assess* Daodi* attributes. These factors are closely related to med-material production and effective ingredient identification, yet these factors are incapable of providing direct evidence to demonstrate safety and effectiveness. Some studies applied modern biomedicine or biostatistics methods for quantity analysis to compare the two types of med-material, even though there is still no consensus of comparison criteria between* Daodi* and non-*Daodi* medicinal material. Until now, only a few studies were conducted through animal experiments and clinical trials to discern the superiority of* Daodi*. These results highly suggested that clinical trials and fundamental studies are needed to explore the effectiveness and safety profiles as well as to further translate the benefits of clinical Chinese medicine into practice.

## Figures and Tables

**Table 1:** Basic information of the included studies.

Category	Characteristic	Number (%) of studies, *n* = 107
*Year*	1990–1999	3 (2.8%)
	2000–2009	56 (52.3%)
	2010–2014.7	48 (44.9%)

*Ref type*	Journal thesis	67 (62.6%)
	Academic degree dissertation^*∗*^	32 (29.9%)
	Conference paper	5 (4.7%)
	Conference recording	3 (2.8%)

*Title*	Comparison of *Daodi* and non-*Daodi* Medicinal Materials	21 (19.6%)
	Comparison of Different Medicinal Materials	11 (10.3%)
	Others	75 (70.1%)

*Author address*	China Academy of Chinese Medical Sciences	10 (9.3%)
	China Pharmaceutical University	8 (7.5%)
	Henan University of Chinese Medicine	8 (7.5%)
	Beijing University of Chinese Medicine	7 (6.5%)
	Chengdu University of TCM	7 (6.5%)
	Hubei University of Traditional Chinese Medicine	6 (5.6%)
	Peking University	5 (4.7%)
	Chinese Academy of Medical Sciences	4 (3.7%)
	Tsinghua University	4 (3.7%)
	Others	48 (44.9%)

*Funding source*	Natural Science Foundation of China	20 (18.7%)
	National Basic Research Program of China	7 (6.5%)
	National Administration of Traditional Chinese Medicine Fund Projects	5 (4.7%)
	Support fund not indicated	44 (41.1%)
	Others	31 (29.0%)

*Competing interests*	Not mentioned	103 (96.3%)

*Journal (n = 67)*	China Journal of Chinese Materia Medica	16 (23.9%)
	Chinese Pharmaceutical Journal	4 (6.0%)
	Journal of Chinese Medicinal Materials	4 (6.0%)
	Lishizhen Medicine and Materia Medica Research	3 (4.5%)
	Others	40 (59.7%)

^*∗*^Some authors were affiliated with different research institutions, and the first affiliated institution was used; academic degree dissertations were categorized according to the university or college.

**Table 2:** Study content categorization of the included studies.

Category	Characteristic	Number (%) of studies, *n* = 107
*Contents of article *	Clinical trial	1 (0.9%)
	Animal experiment	6 (5.6%)
	GBS and soil physical/chemical properties	2 (1.9%)
	Medicinal composition	24 (22.4%)
	Medicinal composition, GBS, and soil physical-chemical property	15 (14.0%)
	Methodology to ascertain *Daodi*	59 (55.1%)

*Medicinal species*	Atractylodis Rhizoma, Lonicerae Japonicae Flos	13 (12.1%)
	Angelicae Sinensis Radix	9 (8.4%)
	Dioscoreae Rhizoma	8 (7.5%)
	Scutellariae Radix, Achyranthis Bidentatae Radix	7 (6.5%)
	Salviae Miltiorrhizae Radix et Rhizoma	6 (5.6%)
	Chuanxiong Rhizoma	5 (4.7%)
	Rehmanniae Radix, Paeonia Lactiflora	4 (3.7%)
	Aconiti Lateralis Radix Praeparata, Alismatis Rhizoma	3 (2.8%)
	Ginseng Radix et Rhizoma, Scrophulariae Radix, Polygalae Radix	2 (1.9%)
	Others	1 (0.9%)

**Table 3:** General information on Chinese traditional medicinal materials.

Category	Characteristic	Number (%) of studies, *n* = 107
Number of the *Daodi* medicinal material original areas	1	44 (41.1%)
	>1	56 (52.3%)
	Not mentioned	7 (6.5%)

Number of the non-*Daodi* medicinal material origin areas	1	10 (9.3%)
	>1	90 (84.1%)
	Not mentioned	7 (6.5%)

Growth mode of the* Daodi* medicinal material	Artificial feeding	41 (38.3%)
	Feral	19 (17.8%)
	Not mentioned	55 (51.4%)

Growth mode of the non-*Daodi* medicinal material^** #**^	Artificial feeding	49 (45.8 %)
	Feral	19 (17.8%)
	Not mentioned	58 (54.2%)

Medicine acquirement	With origin, without source	26 (24.3%)
	With origin and source	84 (78.5%)

Whether it has been identified	Yes	38 (35.5%)
	No	69 (64.5%)

Specific time for reaping	Described	56 (52.3%)
	Not described	51 (47.7%)

Basic property	Described	91 (85.0%)
	Not described	16 (15.0%)

Pharmacological activity and documented efficacy record	Described	89 (83.2%)
	Not described	18 (16.8%)

One study included two med-materials; either the *Daodi *or the *non-Daodi* was produced in more than one region, which resulted in >100%. ^#^Of the basic information, some of the med-materials had been recorded with more than one item about growth mode and material origin, which also produced greater than 100%.

**Table 4:** Ingredients of the studied medicine/soil physical and chemical properties/GBS.

Comparison of medicinal composition, GBS, and soil properties	Number (%) of studies, *n* = 41
*Medicinal composition*	
Inorganic elements	
Element differences	24 (58.5%)
Detection method	22 (53.7%)
Correlation analysis	12 (29.3%)
Accumulation ability	11 (26.8%)
Character index	7 (17.1%)
Others	7 (17.1%)
Organic component	
Active substance	26 (63.4%)
Extracts	5 (12.2%)
Total ash proportion	4 (9.8%)
Polysaccharide	4 (9.8%)
Others	6 (14.6%)
Morphologic	
Physiological anatomy or functional observation	11 (26.8%)
Microanatomy	2 (4.9%)
Ultrastructure	1 (2.4%)
Germplasm resource	7 (17.1%)
Genetic contrast	4 (9.8%)
Endophyte microbes and metabolites	3 (7.3%)
Others	6 (14.6%)
*GBS and soil physical-chemical properties*	
Soil properties	
Inorganic elements	15 (36.6%)
pH	9 (22.0%)
Soil characteristics and type	8 (19.5%)
Available nutrients	7 (17.1%)
Organic material	6 (14.6%)
Physical clay	4 (9.8%)
Soil structure	3 (7.3%)
BS	3 (7.3%)
CEC	3 (7.3%)
Total nutrients	2 (4.9%)
Soil color	2 (4.9%)
Soil moisture	2 (4.9%)
Others	2 (4.9%)
GBS	
Altitude	8 (19.5%)
Latitude	7 (17.1%)
Type of climate or conditions	7 (17.1%)
Annual sunshine duration, rainfall, and temperature	7 (17.1%)
Soil parent material	5 (12.2%)
Topography	4 (9.8%)
Climatic regionalization	4 (9.8%)
Landforms	3 (7.3%)
Vegetation regionalization	3 (7.3%)
Hydrological regionalization	3 (7.3%)
Clay mineral composition	2 (4.9%)
Others	3 (7.3%)

**Table 5:** Methods for assessing *Daodi *medicinal materials.

Methods for assessing *Daodi *medicinal materials	Number (%) of studies, *n* = 59
*Fingerprint*	
Chromatography	
Thin-layer chromatography/TLC	4 (6.8%)
High-performance liquid chromatography (HPLC)	30 (50.8%)
Gas chromatography (GC)	10 (16.9%)
Spectrometry	
Diode array detector (DAD)	8 (13.6%)
Fourier Transform Infrared (FT-IR)	7 (11.9%)
Near Infrared (NIR)	4 (6.8%)
MRI (magnetic resonance imaging)	
Mass spectra (MS)	12 (20.3%)
DNA fingerprint	
Randomly amplified polymorphic DNA (RAPD)	5 (8.5%)
Amplified fragment length polymorphism (AFLP)	1 (1.7%)
Intersimple sequence repeat (ISSR)	1 (1.7%)
Expressed sequence tags-simple sequence repeat (EST-SSR)	1 (1.7%)
Polymerase chain reaction (PCR)	5 (8.5%)
Characteristic fingerprint	21 (35.6%)
*Statistical analysis*	
Cluster analysis	31 (52.5%)
Principal component analysis (PCA)	5 (8.5%)
*Processing methods*	5 (8.5%)
*Others*	7 (11.9%)

**Table 6:** Results and clinical practice.

Characteristic	Number (%) of studies, *n* = 107
*Results*	
*Daodi* medicinal materials were better than non-*Daodi* medicinal materials	69 (64.5%)
No difference between *Daodi* medicinal material and non-*Daodi* medicinal material	2 (1.9%)
*Non-Daodi* medicinal materials were better than *Daodi* medicinal materials	3 (2.8%)
No clear comparison	33 (30.8%)
*Use in clinical practice*	
Having a significant use for clinical practice after finding that the genuine material is distinguishable from the nongenuine material and confirming the quality for the GAP (good agricultural practice) system	98 (91.6%)
Not mentioned	9 (8.4%)
